# Trends and determinants of GPs’ work hours in the UK: a quantitative study

**DOI:** 10.3399/BJGPO.2022.0173

**Published:** 2023-09-20

**Authors:** Alberto Núñez-Elvira

**Affiliations:** 1 National Institute for Health and Care Research Patient Safety Translational Research Centre, Institute of Global Health Innovation, Imperial College London, London, UK

**Keywords:** physicians, general practice, primary health care, hours of work, childbearing

## Abstract

**Background:**

Information on the hours of work of UK doctors is limited, and what exists relies on self-designed questionnaires in England.

**Aim:**

To understand trends in the annual stock of physicians’ hours and their main determinants.

**Design & setting:**

A quantitative study in which data were collected from the Quarterly Labour Force Survey (QLFS) between 1998 and 2020, under the End User Licence (EUL), in the UK.

**Method:**

Descriptive and linear regression models of labour supply for doctors (pooled), GPs, and hospital doctors.

**Results:**

Between 1998 and 2020, while the headcount of doctors grew by 128.79% for hospital doctors and 45.28% for GPs, hours of work dropped by 20.80% for hospital doctors and 25.37% for GPs. Hence, the annual stock of hours grew by 81.20% for hospital doctors but by a modest 8.42% for GPs. Female doctors worked 8.68 fewer hours than males, with GPs reporting the largest reduction (–11.82 hours, 95% confidence interval [CI] = –13.31 to –10.33 and –6.75, 95% CI = –9.32 to –4.19, in the full specification). Family decisions are associated with a fall in doctors’ work hours and modest growth in the annual stock of hours. These determinants and overtime are drivers of part-time work.

**Conclusion:**

Despite the increase in the headcount of GPs, their hours of work dropped over the study period, generating a more steady and modest growth in their total annual stock of hours compared with hospital doctors. Female GPs work fewer hours than male GPs and are more likely to work part-time due to childbearing, marriage/co-habitation, and overtime work.

## How this fits in

Evidence on doctors’ work hours is limited, and what exists in the UK does not track trends over time or compare with headcount growth. This study showed that while the headcount of GPs grew by 45.28% between 1998 and 2020, average weekly work hours fell by 25.37% and their annual stock of hours grew by 8.42%. Female doctors worked, on average, 8.68 fewer hours than male doctors, and female GPs worked 11.82 fewer hours than male GPs. Overtime work, marriage/co-habitation, and childbearing increase the likelihood of working part-time for female GPs compared with female hospital doctors. Indeed, female salaried GPs are most likely to work part-time when their children are aged <5 years.

## Introduction

There is a longstanding concern regarding the shortage of health workers in most health systems,^1^ and this shortfall has been intensified for doctors and nurses.^2,3^ To address these problems, policymakers usually opt to recruit more workers or increase the number of medical students.^4–6^ In 2015, the UK Government announced a plan to recruit 5000 new GPs by 2020, but the success of this plan was questioned because GP full-time equivalents (FTEs) continued to fall between 2016 and 2018.^7^ To counterbalance this drop, the Medical Schools Council has proposed to expand the number of medical school intakes by 5000 per year, reaching 14 500 new graduates in medicine each year.^6,8,9^ However, since training doctors is expensive and takes between 10 and 15 years, the success of such a policy has been questioned in the medical literature.^10,11^

The Health and Social Care Committee has recently outlined the main challenges faced by the healthcare sector in obtaining a more sustainable workforce.^8^ However, in general practice, the situation requires a broader plan to address the current workforce crisis.^9^ Developing this strategy requires a better understanding of what is needed for supply and demand.^12^ One of the main weaknesses of analyses from the supply side is that they are only focused on how many doctors the UK needs, and only deal with headcounts (number of workers) and FTEs. Although the FTE measure may provide some information on full-time roles, these supply-side analyses refrain from examining work hours more extensively. A better understanding of the trends and determinants of work hours is essential for improving doctors’ labour supply decisions and workforce planning.

Despite their importance, there is little evidence on doctors’ work hours, and what exists in the literature is more focused on hospital doctors.^13–15^ For GPs, evidence is even more limited and mostly restricted to Canada.^16,17^ For the UK, existing evidence acknowledges differences in the work hours of English GPs compared with other occupations^18^ but usually relies on self-designed surveys.^19,20^

This study provides new evidence for the UK between 1998 and 2020. Using data from the QLFS, this study combines headcount figures from the Review Body on Doctors' and Dentists' Remuneration (DDRB) to examine trends in the hours of work of UK doctors (GPs and hospital doctors). This will improve the understanding of the trends in work hours and the annual stock of hours supplied by doctors. With the increase in numbers of women working in the medical profession,^21,22^ this study estimates hours of work at the individual level to ascertain how decisions such as marriage/co-habitation, childbearing, or overtime may impact doctors’ decisions to supply more or fewer hours. This can be relevant for workforce planners and health managers.

## Method

A unique dataset was obtained by pooling quarterly cross-sectional datasets between 1998 and 2020 from the QLFS under the EUL. The QLFS tracks socioeconomic changes in the UK labour market characteristics^23^ and reports valuable information quarterly, covering approximately 40 000 private households and >600 variables. It spans a long range of years and variables to address regional and country differences within the UK.^24^ Since 1993, the data have been collected in five consecutive waves. Each wave corresponds to 13 weeks that corresponds to winter, spring, summer and autumn seasons. Every wave, 20% of the sample is replaced. The response rate varied among the five waves but remained at nearly 40% overall at the minimum. Between 2011 and 2019, the average response rate decreased from 50% in 2011 to 36% in 2019.^25^ In most cases, the response rate in wave 1 was above 50%. For example, between October and December 2019, the wave 1 response rate was 54%,^25^ and nearly 70% for the same wave and period in 2006.^26^

The QLFS provides an exceptional data source, but it is underexplored for the analysis of UK doctors’ labour supply. Doctors were identified using Standard Occupation Classification (SOC). The breakdown into GPs and hospital doctors was obtained by combining SOC codes with Standard Industrial Classification (SIC). The QLFS also enables the breakdown of GPs into self-employed and employed to distinguish between partnered and salaried GPs.

The analysis relies on a self-constructed variable of total hours of work per week, which comprises usual hours in the main job (basic and paid or unpaid overtime) and actual hours worked in any second job. The basic usual hours of work are derived from age, overtime work (EVEROT), total usual hours of work (TOTUS), and usual hours of work (USUHR). The hours worked in the second job are independent and comprise paid and unpaid overtime. This information has been consistently reported every quarter and year. In 2018, the following questions were asked:

- *How many hours per week do you usually work in your (main) job or business — please exclude meal breaks?* (TOTUS1)
- *Thinking of your (main) job or business, how many hours per week do you usually work — please exclude meal breaks and overtime?* (USUHR)
- *Do you ever do any work that you could regard as paid or unpaid overtime?* (EVEROT)
- *How many hours did you actually work in the week ending Sunday the* [ref date] *in your second job in total, including any paid or unpaid overtime — please exclude meal breaks?* (ACTHR2)

This study gathered data (headcounts) from the DDRB between 1998 and 2020,^27,28^ and computed doctors’ average total weekly work hours for each year by sex. The annual stock of hours supplied by GPs and hospital doctors was calculated by multiplying the average work hours obtained from the QLFS by the headcount figures from the DDRB reports from the Office of Manpower Economics and then multiplying by 52 weeks. Kernel densities were used to plot the distribution of work hours.^29^ Linear regression models were used to estimate work hours for the following four main groups: GPs; hospital doctors; GP partners; and salaried GPs, controlling for sex, age, and the interaction between sex and other controls. A final analysis computed the probability of working part-time (probit) and their marginal effects to estimate potential sex preferences for part-time work, and to what extent. This could be important for identifying the main drivers of the supply of hours. Potential heterogeneity issues were addressed by controlling for the regional fixed effects.

The analysis describes trends in headcount and work hours since 1998, when the European Working Time Directive (EWTD) was enacted in the UK, although it was passed in 1993. Full compliance with NHS workers was agreed on by August 2009.^15^ It restricts working hours to an average of 48 hours per week over a 17-week period. The year 2004 was considered the reference year for two main reasons. First, doctors agreed on a new contract in 2004. Second, EWTD was fully implemented for NHS workers in August 2009.

## Results

Table 1 reports the main descriptive statistics for 1998–2020. The GPs accounted for 35.29% of the total sample size (see Supplementary Table S1). In total, 70.23% of the GPs were GP partners. The proportion of female doctors was above 40% and close to that of the general population; for example, between 2009 and 2017, the proportion of females found in the sample was 44.52% (see Supplementary Table S2) versus 45% reported by NHS Digital,^30^ and, in 2018, 46.02% in the sample (see Supplementary Table S3) versus 47%.^31^ The proportion of female doctors was greater among GPs (46.21%) than hospital doctors (39.74%) (see Supplementary Table S2). The average total hours of work reported between 1998 and 2020 was 44.66 hours per week overall (95% CI = 44.46 to 44.86), 40.60 hours for GPs (95% CI = 40.24 to 40.96), and 46.87 hours for hospital doctors (95% CI = 46.63 to 47.11). Female GPs worked 10.99 hours fewer than males; 34.69 (95% CI = 34.24 to 35.14) hours per week versus 45.68 (95% CI = 45.18 to 46.18) hours. Female hospital doctors worked more hours than GPs but still fewer than males (–6.12 hours), averaging 43.18 (95% CI = 42.82 to 43.55) hours per week compared with 49.30 (95% CI = 48.99 to 49.61) hours for male hospital doctors.

**Table 1.** Sample characteristics (1998–2020)

	Pooled		GPs		Hospital doctors	
Variables	Mean	SD	Mean	SD	Mean	SD
*n*	24 519		8652		15 867	
Total hours in main and second job	44.66	16.24	40.60	17.01	46.87	15.37
Part-time	0.19	0.39	0.32	0.47	0.12	0.33
GP	0.35	0.48	1.00	0.00		
GP partner	0.25	0.43	0.70	0.46		
GP salaried	0.11	0.31	0.30	0.46		
Hospital doctor	0.65	0.48			1.00	0.00
Employee (salaried)	0.74	0.44	0.30	0.46	0.97	0.16
Age, years	42.75	10.68	45.66	10.79	41.16	10.28
Female	0.42	0.49	0.46	0.50	0.40	0.49
Ever worked overtime	0.52	0.50	0.36	0.48	0.61	0.49
Second job	0.14	0.34	0.15	0.36	0.13	0.33
Public sector	0.86	0.35	0.66	0.47	0.97	0.18
Work experience	10.25	9.42	12.72	10.14	8.90	8.70
Married	0.85	0.36	0.89	0.32	0.83	0.37
Children aged <5 years	0.24	0.43	0.22	0.41	0.25	0.43
Children aged 5–9 years	0.13	0.34	0.13	0.33	0.13	0.34
Children aged 10–15 years	0.13	0.33	0.16	0.37	0.11	0.32
No children aged <16 years	0.50	0.50	0.50	0.50	0.51	0.50
White	0.71	0.45	0.81	0.39	0.66	0.47
Asian	0.20	0.40	0.13	0.34	0.23	0.42
Black	0.03	0.17	0.01	0.12	0.04	0.19
Other than White, Asian, or Black	0.00	0.00	0.00	0.00	0.00	0.00
UK or British	0.68	0.47	0.79	0.41	0.63	0.48

SD = standard deviation.

**Figure 1.**
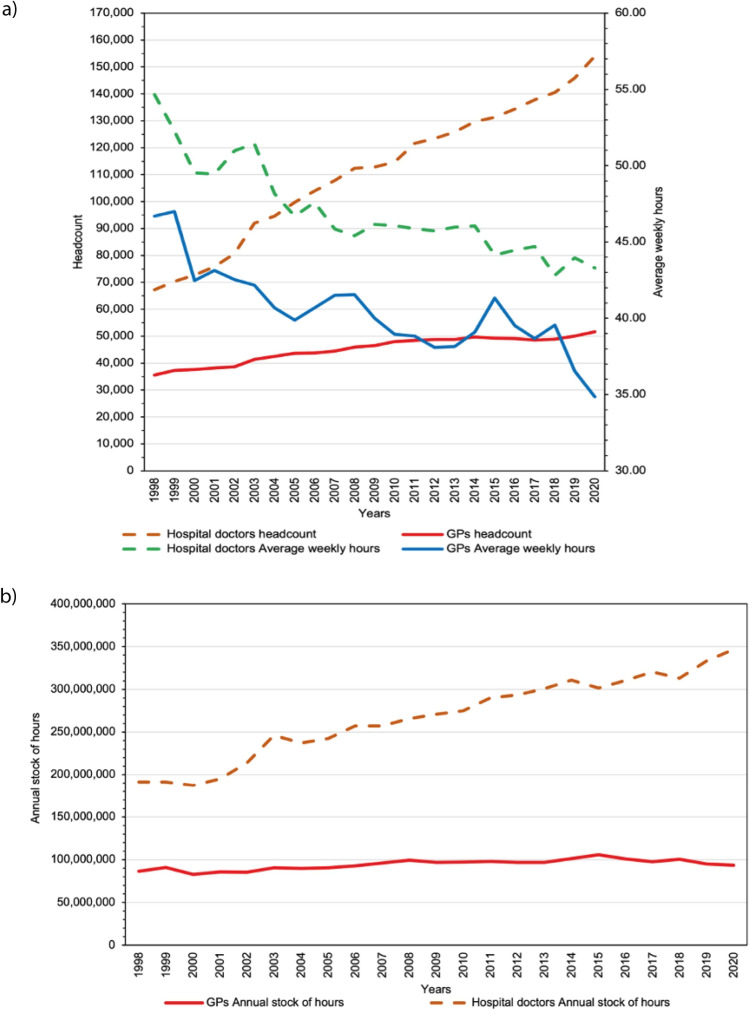
a) Headcount and average weekly total work hours by year; and b) annual stock of hours

Figure 1 tracks trends in the headcount and average weekly total work hours by year (Figure 1a), and the annual stock of hours (Figure 1b). Headcounts increased by 128.79% for hospital doctors and by 45.28% for GPs (see Supplementary Table S4). However, there has been a downward trend in the average weekly total work hours, which is more accentuated for hospital doctors (–0.41 hours per year) than for GPs (–0.35 hours per year) (Figure 1). Between 1998 and 2020, hours dropped by 20.80% for hospital doctors and –25.37% for GPs and the total annual stock of hours supplied increased by 81.20% for hospital doctors and by a modest 8.42% for GPs.

**Figure 2.**
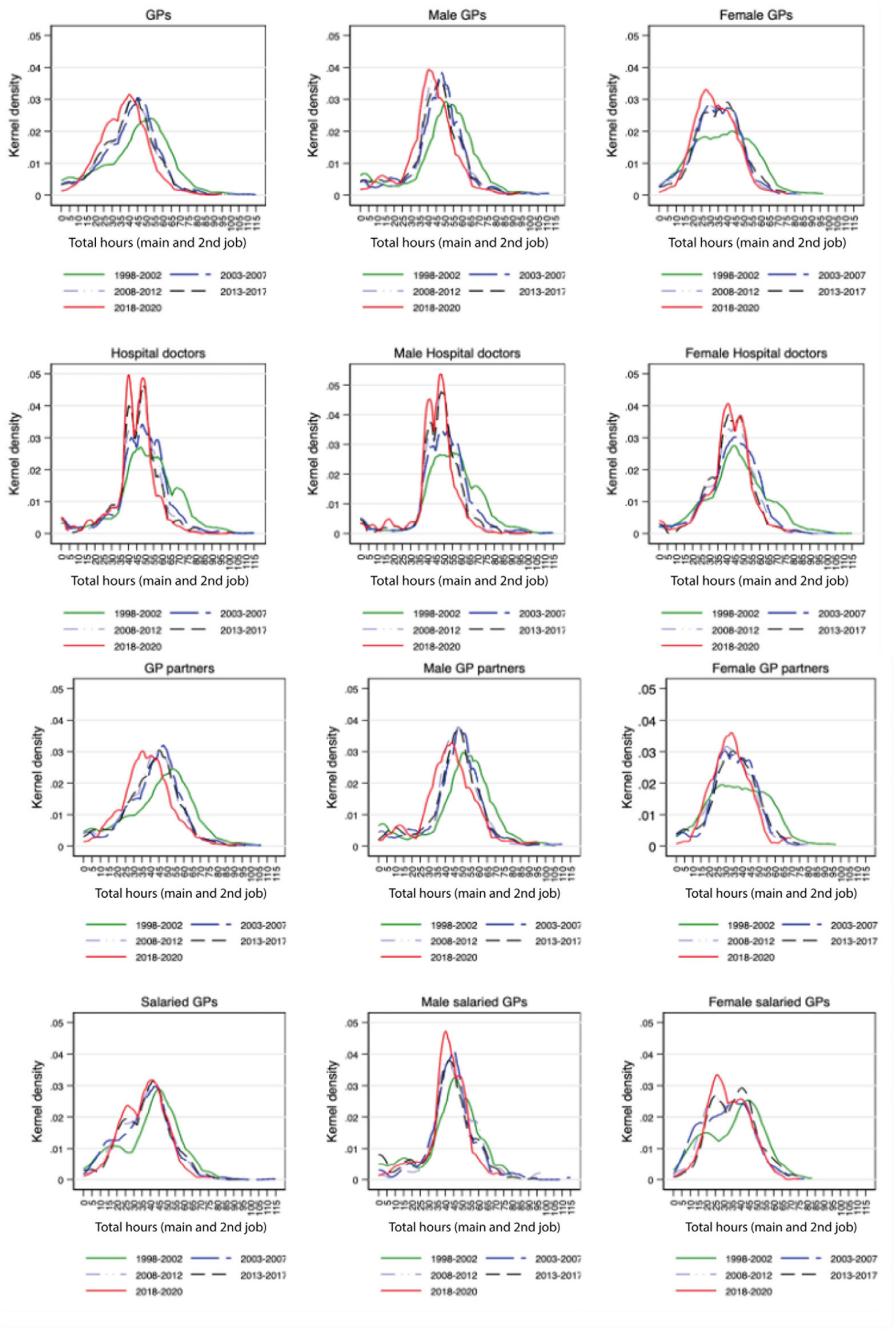
Kernel distributions of total hours of work in 5-year periods

Figure 2 portrays the sex distribution of total hours in 5-year intervals to identify when the fall in hours occurred and whether it was attributed to changes in the sex proportion of doctors. The proportion of female GPs increased from 50% in 2015 to 56% in 2020, and 41% in 2009 to 45% for hospital doctors in 2017.^32^ The kernel densities show that the reduction in average weekly hours is larger for GPs than for hospital doctors and larger for female workers, with the largest reduction for female salaried GPs. The large density of female workers below the FTE level (37.5 hours for GPs and 40 hours for hospital doctors) shows that their proportion working part-time is larger than that of hospital doctors.

Table 2 presents the estimates obtained from the regression models. The full table is provided in Supplementary Table S5. Supplementary Table S6 contains estimates with the dependent variables transformed into logarithms. On average, GPs worked fewer hours than hospital doctors (–3.69 hours, 95% CI = −4.64 to −2.74). Female doctors worked –8.68 (95% CI = –9.92 to –7.43) fewer hours than males (Model 1), while female GPs showed the highest fall in hours (–11.82, 95% CI= −13.31 to –10.33 in Model 1 and –6.75, 95% CI = −9.32 to −4.19 in Model 2). Hence, female GPs worked about 3.14 fewer hours compared to female doctors in the pooled model (Model 1). Some year estimates were significant (2003 and 2018). Lifecycle decisions (marriage/co-habitation or children) largely impacted the supply of hours (Model 2) for married/co-habiting female GPs (–7.02, 95% CI = −10.44 to –3.60) and married/co-habiting female partner GPs (–10.16, 95% CI = −15.82 to –4.50). Having children reduced work hours and it was greatest when children were aged <5 years in all occupations (Model 1), with salaried GPs reporting the largest reduction (–6.48, 95% CI = −9.03 to –3.92). Interacting these variables with sex results in a drop for females, with hospital doctors showing the largest fall.

**Table 2.** Ordinary least squares estimates for total hours in main and second job (1998–2020)

	Model 1: basic specification					Model 2: full model				
Category	Pooled	GPs	GP partners	GP salaried	Hospital doctors	Pooled	GPs	GP partners	GP salaried	Hospital doctors
**Female**	–8.68^a^	–11.82^a^	–12.49^a^	–9.45^a^	–6.89^a^	–0.21	–6.75^a^	–2.76	–9.49^b^	3.26
	(0.58)	(0.69)	(0.60)	(1.21)	(0.52)	(1.53)	(1.19)	(1.76)	(2.89)	(3.52)
**GP**	–3.69^a^	—	—	—	—	–1.03	—	—	—	—
	(0.44)	—	—	—	—	(0.54)	—	—	—	—
**UK or British**	–1.07	–2.64^c^	–2.00	–3.71^c^	–0.35	–0.42	–2.77^c^	–2.54	–1.71	0.78
	(0.50)	(1.01)	(1.06)	(1.55)	(0.41)	(0.47)	(1.08)	(1.39)	(1.03)	(0.47)
**Married or co-habiting**	–1.15	–0.25	–0.75	–0.38	–1.58^c^	0.06	5.08^a^	7.14^b^	3.19	–1.43
	(0.59)	(0.77)	(0.71)	(1.36)	(0.65)	(0.91)	(1.10)	(1.91)	(2.20)	(1.09)
**Children aged <5 years**	–3.51^a^	–4.65^a^	–2.97^c^	–6.48^a^	–2.79^a^	0.14	–1.74	–1.12	–3.57	0.67
	(0.61)	(0.91)	(1.21)	(1.18)	(0.64)	(0.63)	(1.05)	(1.78)	(2.40)	(0.69)
**Children aged 5–9 years**	–2.29^b^	–2.93^c^	–1.34	–5.25^c^	–1.95	0.70	–0.66	0.87	–5.75	0.98
	(0.66)	(1.01)	(1.16)	(2.11)	(0.95)	(0.47)	(1.56)	(1.73)	(2.98)	(0.91)
**Children aged 10–15 years**	–0.45	0.44	1.50	–1.73	–1.08	1.99^a^	1.93	2.88	–2.14	1.51
	(0.41)	(1.10)	(1.44)	(1.14)	(0.68)	(0.35)	(1.29)	(1.37)	(2.28)	(0.80)
**GP x female**	—	—	—	—	—	–4.68^a^	—	—	—	—
	—	—	—	—	—	(0.71)	—	—	—	—
**UK or British x female**	—	—	—	—	—	–1.82^b^	1.03	2.76	–2.92	–2.98^a^
	—	—	—	—	—	(0.51)	(1.31)	(1.48)	(2.54)	(0.63)
**Married or co-habiting x female**	—	—	—	—	—	–1.39	–7.02^a^	–10.16^b^	–4.73	0.15
	—	—	—	—	—	(0.93)	(1.58)	(2.62)	(2.57)	(1.35)
**Children aged <5 years x female**	—	—	—	—	—	–8.35^a^	–6.13^b^	–5.66	–4.55	–8.46^a^
	—	—	—	—	—	(1.09)	(1.60)	(3.31)	(3.54)	(1.02)
**Children aged 5–9 years x female**	—	—	—	—	—	–7.39^a^	–5.38^c^	–6.60^c^	1.05	–7.69^a^
	—	—	—	—	—	(0.86)	(2.07)	(2.44)	(3.00)	(0.79)
**Children aged 10–15 years x female**	—	—	—	—	—	–6.12^a^	–3.52	–4.48^c^	1.76	–7.26^a^
	—	—	—	—	—	(0.69)	(1.77)	(1.77)	(3.99)	(1.22)
**Constant**	49.77^a^	47.33^a^	48.35^a^	41.50^a^	40.67^a^	44.82^a^	42.67^a^	40.61^a^	40.65^a^	36.86^a^
	(1.17)	(1.50)	(2.09)	(2.34)	(2.41)	(1.34)	(1.29)	(1.70)	(2.49)	(3.20)
***n***	23 551	8281	5772	2509	15 270	23 551	8281	5772	2509	15 270
***R* ^2^**	0.249	0.315	0.339	0.268	0.201	0.275	0.339	0.362	0.313	0.224
**Adjusted *R* ^2^**	0.247	0.309	0.332	0.249	0.198	0.272	0.331	0.352	0.288	0.220

^a^ *P*<0.001. ^b^ *P*<0.01. ^c^ *P*<0.05. Robust standard errors in parentheses were adjusted for clustering at the regional level. The dependent variable is the total hours worked in the main and second job.

The literature reports that a higher proportion of females work part-time.^21,22^ Table 3 summarises the marginal effects of the probit model; see Supplementary Table S7 for 95% CIs. Covariates that increase the likelihood of working part-time are sex (female), having children aged <5 or 5–9 years, or working overtime. Females working overtime (Model 2) were more likely to work part-time, with female GPs being more likely (0.08, 95% CI = 0.04 to 0.11) than hospital doctors (0.03, 95% CI = 0.01 to 0.04); salaried GPs showed the largest coefficient (0.14, 95% CI = 0.04 to 0.24). Married/co-habiting female GPs were 16% (95% CI = 0.04 to 0.29) more likely to work part-time than hospital doctors (6%, 95% CI =−0.00 to 0.13); salaried GPs showed the largest coefficient (22%, 95% CI = 0.06 to 0.38). This also holds true when female GPs have children aged <5 years, especially female salaried GPs (0.35, 95% CI = 0.15 to 0.54), but with large variation.

**Table 3.** Marginal effects of the probability of working part-time (1998–2020)

	Model 1: basic specification					Model 2: full model				
Category	Pooled	GPs	GP partners	GP salaried	Hospital doctors	Pooled	GPs	GP partners	GP salaried	Hospital doctors
**Female**	0.27^a^	0.36^a^	0.35^a^	0.37^a^	0.21^a^	–0.03	0.13^b^	0.12	0.13	–0.05
	(0.01)	(0.02)	(0.01)	(0.02)	(0.01)	(0.04)	(0.06)	(0.09)	(0.15)	(0.05)
**GP**	0.10^a^	0.00	0.00	0.00	0.00	0.06^a^	0.00	0.00	0.00	0.00
	(0.01)	(.)	(.)	(.)	(.)	(0.02)	(.)	(.)	(.)	(.)
**UK or British**	0.07^a^	0.08^b^	0.06^b^	0.12^b^	0.05^c^	0.05^b^	0.06	0.06	0.05	0.03
	(0.02)	(0.03)	(0.03)	(0.06)	(0.02)	(0.02)	(0.04)	(0.05)	(0.04)	(0.02)
**Married or co-habiting**	0.04^b^	0.06	0.10^b^	0.02	0.04	–0.04	–0.09	–0.05	–0.16	–0.02
	(0.02)	(0.03)	(0.04)	(0.08)	(0.02)	(0.03)	(0.06)	(0.08)	(0.09)	(0.03)
**Children aged <5 years**	0.13^a^	0.13^a^	0.08^c^	0.21^a^	0.12^a^	–0.03^c^	–0.05	–0.02	–0.08	–0.03^b^
	(0.01)	(0.02)	(0.03)	(0.03)	(0.01)	(0.01)	(0.04)	(0.02)	(0.09)	(0.01)
**Children aged 5–9 years**	0.08^a^	0.0^a^	0.04	0.11^c^	0.08^a^	–0.05^b^	–0.05	–0.11	0.10	–0.04
	(0.02)	(0.02)	(0.02)	(0.04)	(0.02)	(0.02)	(0.06)	(0.09)	(0.07)	(0.02)
**Children aged 10–15 years**	0.05^a^	0.01	–0.01	0.03	0.07^a^	–0.05^c^	–0.09^b^	–0.09^c^	–0.15	–0.03
	(0.01)	(0.02)	(0.02)	(0.05)	(0.01)	(0.02)	(0.04)	(0.03)	(0.08)	(0.02)
**Overtime work**	–0.06^a^	–0.05^a^	–0.04^c^	–0.08^b^	–0.06^a^	–0.09^a^	–0.10^a^	–0.07^c^	–0.18^a^	–0.07^a^
	(0.01)	(0.01)	(0.01)	(0.03)	(0.01)	(0.01)	(0.02)	(0.03)	(0.04)	(0.01)
**Second job**	0.07^a^	0.12^a^	0.10^a^	0.19^a^	0.04^c^	0.10^a^	0.21^a^	0.17^a^	0.32^a^	0.04^b^
	(0.01)	(0.02)	(0.02)	(0.04)	(0.01)	(0.01)	(0.02)	(0.02)	(0.04)	(0.02)
**GP x female**	—	—	—	—	—	0.05^c^	0.00	0.00	0.00	0.00
	—	—	—	—	—	(0.02)	(.)	(.)	(.)	(.)
**UK or British x female**	—	—	—	—	—	0.01	0.00	–0.03	0.06	0.02
	—	—	—	—	—	(0.02)	(0.03)	(0.05)	(0.06)	(0.02)
**Married or co-habiting x female**	—	—	—	—	—	0.09^a^	0.16^b^	0.15	0.22^c^	0.06
	—	—	—	—	—	(0.03)	(0.06)	(0.08)	(0.08)	(0.03)
**Children aged <5 years x female**	—	—	—	—	—	0.22^a^	0.26^a^	0.16^c^	0.35^a^	0.20^a^
	—	—	—	—	—	(0.02)	(0.06)	(0.06)	(0.10)	(0.02)
**Children aged 5–9 years x female**	—	—	—	—	—	0.19^a^	0.18^c^	0.22^b^	–0.01	0.18^a^
	—	—	—	—	—	(0.03)	(0.06)	(0.10)	(0.07)	(0.02)
**Children aged 10–15 years x female**	—	—	—	—	—	0.16^a^	0.14^a^	0.13^c^	0.19	0.16^a^
	—	—	—	—	—	(0.02)	(0.04)	(0.05)	(0.10)	(0.02)
**Overtime work x female**	—	—	—	—	—	0.04^a^	0.08^a^	0.06	0.14^c^	0.03^c^
	—	—	—	—	—	(0.01)	(0.02)	(0.03)	(0.05)	(0.01)
**Second job x female**	—	—	—	—	—	–0.04	–0.14^a^	–0.12^a^	–0.19^b^	0.01
	—	—	—	—	—	(0.02)	(0.03)	(0.03)	(0.08)	(0.02)
***n***	23 526	8278	5770	2507	15 248	23 526	8278	5770	2502	15 248

^a^ *P*<0.001. ^b^ *P*<0.05. ^c^ *P*<0.01. Robust standard errors in parentheses were adjusted for clustering at the regional level. The dependent variable is part-time, which takes value one if individual working part-time and zero otherwise. Full table can be found in Supplementary Table S7. (.) = missing data because that variable was not included in the model.

## Discussion

### Summary

This study examined trends in doctors’ work hours in the UK between 1998 and 2020. While the headcounts of hospital doctors grew by 128.79% and 45.28% for GPs, their average weekly work hours fell by 20.80% and 25.37%, respectively. The overall change in the annual stock of hours grew by 81.20% for hospital doctors and by 8.42% for GPs. This study also examined the main determinants of doctors’ work hours since the growing feminisation of the medical workforce. This is important, because it has been assumed that female doctors may have increased their part-time work. The study found that female doctors worked 8.68 fewer hours than males, and female GPs worked even 3.14 fewer hours (95% CI = −3.41 to –2.90). Childbearing and marriage/co-habitation reduced the hours supplied by females, especially when their children are aged <15 years. Overtime hours also influenced female doctors’ decisions regarding part-time work.

### Strengths and limitations

The main strength of this study lies in the data source. The QLFS provides an excellent framework because it has the largest sample size from UK households, with relatively small sampling errors owing to the different waves that build up the survey, and a large range of variables from the labour market included in each quarter. The sample size is sufficiently large with relevant variables from the labour market.

However, this study has some limitations. First, the QLFS is not based on the population of UK doctors, but only on the characteristics of the UK population. In addition, it only permits the breakdown of GPs into partners and salaried, and there is no information on specialties for hospital doctors. In the early years, hours of work had much variability, especially for salaried GPs owing to small sample sizes. This study refrains from controlling for wages for the following reasons. First, wages are endogenous to hours of work and may influence doctors’ decisions regarding their work hours. Second, the QLFS does not incorporate earnings information for self-employed workers (GP partners). If the study controlled for wages, GP partners would be excluded from the analysis, resulting in an incomplete analysis.

### Comparison with existing literature

Labour supply analyses corroborate a fall in hours of work over time.^33^ Previous studies on doctors have focused on examining the determinants for hospital doctors^14,34^ or GPs.^16,17^ In the UK, evidence has focused on GPs without using information from publicly available surveys, such as the QLFS.^18^ Similar conclusions were reached in this study. The estimate for 2003 might reflect the anticipated impact of the new contract in 2004.

Most studies agreed on the existence of gender differences in the supply of hours for certain specialties.^21,22,35^ This study goes beyond and provides the most comprehensive framework for the analysis of the main issues regarding the labour supply of doctors in the UK. First, the analysis covered GPs and hospital doctors, breaking down GPs into partners and salaried GPs. One of the most discussed topics deals with gender differences in the supply of hours of work but most evidence does not find substantial differences.^21,22,36^ Nevertheless, existing evidence for England found that female GPs worked 11.8 hours fewer than male GPs and that female GPs reported higher proportions in part-time work.^18^ The main findings have corroborated that female doctors work fewer hours than males (8.68 fewer hours), and female GPs work 11.82 hours fewer than male GPs. The findings have also quantified how children impact these decisions, which has coincided with the results of previous studies with their own surveys.^16,18,37,38^

### Implications for practice

This work is important for policymakers to improve the training, recruitment, and retention of UK doctors. This study has confirmed that efforts should be made to address female doctors to reduce their proportion of working part-time and increase the number of FTEs. Designing new incentives must focus on reducing the overtime burden and improving the work–life balance for childbearing.

## Data

Please refer to the UK Data Service website data catalogue for the Quarterly Labour Force Survey serial numbers from January–March 1998 to October–December 2020.
